# Evaluating Methods for Aflatoxin B1 Monitoring in Selected Food Crops Within Decentralized Agricultural Systems

**DOI:** 10.3390/toxins17010037

**Published:** 2025-01-14

**Authors:** Haadia Tanveer, Hannah Glesener, Blake Su, Brooke Bolsinger, Rosa Krajmalnik-Brown, Lee E. Voth-Gaeddert

**Affiliations:** 1 Biodesign Center for Health Through Microbiomes, Arizona State University, Tempe, AZ 85281, USA; htanveer@asu.edu (H.T.); glesener@asu.edu (H.G.); dr.rosy@asu.edu (R.K.-B.); 2 School for Life Sciences, Arizona State University, Tempe, AZ 85281, USA; 3 School for Engineering of Matter, Transport, and Energy, Arizona State University, Tempe, AZ 85281, USA; 4 School of Computing and Augmented Intelligence, Arizona State University, Tempe, AZ 85281, USA; 5 School of Sustainable Engineering and the Built Environment, Arizona State University, Tempe, AZ 85281, USA; 6 Center for Indigenous Health Research, Wuqu’ Kawoq | Maya Health Alliance, Tecpan, Chimaltenango 04006, Guatemala

**Keywords:** mycotoxins, maize, surveillance, aspergillus, subsistence farming

## Abstract

Aflatoxin B1 (AFB1) contamination of food crops pose severe public health risks, particularly in decentralized agricultural systems common in low-resource settings. Effective monitoring tools are critical for mitigating exposure, but their adoption is limited by barriers such as cost, infrastructure, and technical expertise. The objectives of this study were: (1) to evaluate common AFB1 detection methods, including enzyme-linked immunosorbent assays (ELISA) and lateral-flow assays (LFA), validated via high-performance liquid chromatography (HPLC), focusing on their suitability for possible applications in decentralized, low-resource settings; and (2) to conduct a barriers-to-use assessment for commonly available AFB1 detection methods and their applicability in low-resource settings. Among four ELISA kits, the AgraQuant Aflatoxin B1 2/50 ELISA Kit demonstrated the highest accuracy and precision, reliably quantifying AFB1 in maize and tortillas across 5–150 ppb with minimal cross-reactivity. For LFA, a smartphone-based algorithm achieved a high presence/absence accuracy rate of 84% but struggled with concentration prediction. The barriers-to-use analysis highlighted the practicality of low-cost tools like moisture readers for field screening but underscored their qualitative limitations. Advanced methods like HPLC and LC-MS offer greater precision but remain impractical due to their high costs and infrastructure requirements, suggesting a potential role for adapted ELISA or LFA methods as confirmatory approaches. These findings support the development of multi-tiered frameworks integrating affordable field tools with regional or centralized confirmatory testing. Addressing systemic barriers through capacity building, partnerships, and improved logistics will enhance AFB1 monitoring in decentralized systems, protecting public health in vulnerable communities.

## 1. Introduction

Aflatoxins are mycotoxins, or fungal metabolites, produced primarily by the *Aspergillus flavus* and *Aspergillus parasiticus* species. The four major aflatoxins are aflatoxins B_1_, B_2_, G_1_, and G_2_ (AFB1, AFB2, AFG1, and AFG2), among which AFB1 is the most toxic [1]. AFB1 is linked to hepatocellular carcinoma, acute aflatoxicosis, immune suppression, malnutrition, and growth impairments [2,3]. Aflatoxins are primarily found in maize, other grains, oilseeds, spices, and tree nuts, while other products like milk, meat, and eggs can become contaminated via animal feed [2]. This widespread contamination of the global food supply poses a major risk of chronic aflatoxin exposure to approximately 4.5 billion people, with low-resource regions and decentralized agricultural systems bearing a disproportionate burden [1]. This is often due to limited or costly testing methods, shorter supply chains between farmer and consumer, informal markets, and weak regulatory frameworks [4]. Additionally, low-resource populations where maize is a dietary staple, face particularly compounded risks [5,6,7]. These populations face challenges with decentralized, smallholder farming practices and supply chains, environmental sanitation, informal food storage, and limited food regulation enforcement along with favorable conditions for fungal growth like warm temperatures and high humidity [2,8].

Throughout the maize supply chain, many points of potential aflatoxin contamination arise due to pre-harvest and post-harvest factors including agronomic and agricultural practices, transportation, storage, and processing methods [9]. These supply chains, which coordinate the distribution of maize to consumers, begin with production by farmers but may then include (1) direct-to-consumer distribution (households or restaurants), (2) local co-op grain silos, (3) logistics and shipping companies (often connecting co-ops to processing facilities), or (4) food processing facilities. Regulatory and monitoring processes can vary based on the structure of the supply chains, since more centralized supply chains offer fewer and more efficient monitoring points. Decentralized supply chains are often associated with distributed and informal production, logistics, and marketing, which reduce efficiency of monitoring points. Furthermore, the implementation of food regulations for aflatoxins is important, often setting limits to aflatoxin concentrations (e.g., 20 parts per billion, ppb, in the United States) in foods intended for human consumption [10]. However, given the complicated nature of both centralized and decentralized supply chains and potential points of contamination and monitoring, understanding effective testing methods is critical. Specifically, the deployment of effective AFB1 testing methods for maize in resource-limited or decentralized settings is essential to promote agricultural best practices and prevent the adverse human health effects associated with AFB1 exposure.

In the United States, where centralized supply chains are prevalent, the Federal Grain Inspection Service (FGIS) is responsible for approving aflatoxin testing methods that adhere to USDA and FDA guidelines. While there are many methods available, FGIS-verified aflatoxin detection methods span spectroscopic, chromatographic, and antibody-based methods. These include high-performance liquid chromatography (HPLC), liquid chromatography/tandem mass spectrometry (LC/MS), enzyme-linked immunosorbent assays (ELISA), and lateral flow assay (LFA) dipsticks [11,12,13]. HPLC and LC-MS methods are the most precise and accurate but are also expensive, time-consuming, and require highly trained technicians. These are barriers to accessibility, especially in low-resource settings [13]. Consequently, alternative testing methods may be advantageous. Among these alternatives, ELISA kits and LFA dipsticks can be used for both on-site (field) or off-site (lab) monitoring of AFB1 in grain and food processing. Additionally, moisture content and the Bright Greenish-Yellow Fluorescence (BGYF) test are indicators of a conducive environment or purported fungal presence, though they are not direct measures of AFB1 presence [14]. These alternative methods to HPLC and LC-MS are relevant to monitor grain storage, to sort aflatoxin-contaminated maize for efficient removal, and to prompt further confirmatory analyses in bulk samples [15,16,17]. These AFB1 detection methods can enhance AFB1 testing throughout the maize supply chain when strategically applied and integrated with pre- and post-harvest mitigation strategies [14]. Table 1 summarizes the various testing methods, application and purpose, supply chain relevance, and pros and cons for each method.

Moisture content analysis and BGYF testing are well suited for indirect monitoring of AFB1 in both high- and low-resource settings, whereas ELISA and LFA approaches could be better integrated to benefit stakeholders within low-resource, decentralized agricultural production and supply chains. In this study, we facilitate (1) an objective comparison of commercial ELISA kits for quantifying AFB1 in maize and food products, and (2) the minimization of costly LFA dipstick digital readers. In addition, (3) we evaluate limitations or barriers-to-use for all listed methods within decentralized, low-resource settings. Results can help practitioners understand challenges and develop tailored monitoring frameworks.

ELISA kits utilize competitive antibody recognition for simple, rapid, and cost-effective aflatoxin quantification. However, USDA regulations focus on total aflatoxins, as opposed to AFB1-specific kits. The specificity of ELISA antibodies to compounds rather than antigens present potential for cross-reactivity with other mycotoxins. However, monitoring AFB1 specifically in low-resource settings may provide benefits by targeting the most toxic and prevalent aflatoxin, allowing for focused risk assessment and mitigation efforts, while offering a cost-effective and scalable option that could simplify testing and interpretation protocols for regions with limited resources and infrastructure [1]. In addition, validation of ELISA in testing AFB1 across a wide variety of matrices is currently lacking [11]. ELISA kits are also implemented in research settings to monitor the efficacy of detoxification strategies. Previous validation studies of ELISA tests often focused on lower detection ranges while naturally occurring AFB1 concentrations reach thousands of ppb requiring protocol augmentation [1].

LFA dipsticks are promising for their simple, rapid on-site detection of AFB1, yet require further improvements in sensitivity and cost-effective reading for AFB1 quantification [12]. Current methods require the use of expensive digital readers that require electrical outlets, which hampers deployment in on-site, off-grid testing. Given advancements in smartphone technology and penetration of use, there are demonstrated LFA readers facilitated via smartphone for similar targets [21]. To demonstrate feasibility, initial testing of image processing and model development are critical.

Finally, there are many general barriers-to-use of these detection methods within decentralized agricultural production and supply chains in low-resource settings. Among these, examples of limitations warranting evaluation include the need for power supply, procurement issues, and other laboratory requirements. Systematically evaluating these barriers can provide a clearer picture to both academic researchers, field practitioners, and policymakers on the most effective approaches to improve feasible use in a wide variety of settings.

In this study, we aim to comparatively evaluate ELISA kits’ quantification of AFB1 contamination of maize and tortillas, as well as identify alternative approaches to digital readers of LFAs by implementing imaging algorithms. Finally, we aim to comprehensively assess the limitations or barriers-to-use of common AFB1 quantification methods in decentralized agricultural systems in low-resource settings. In doing so, our goal is to strengthen options for both researchers and practitioners to employ in monitoring AFB1 in order to reduce human exposure to and consumption of the carcinogen.

## 2. Results

### 2.1. ELISA Results

**Figure 1:**
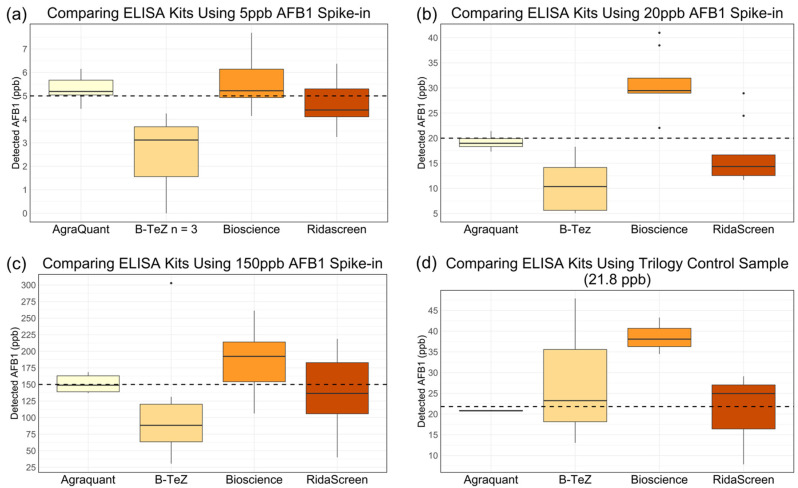
Comparison of ELISA AFB1 Testing Kits at Varying Concentrations. AgraQuant ELISA kit had more accurate recovery with better precision than B-TeZ, Bioscience, and Ridascreen ELISA kits with extracted ground maize samples at (**a**) 5 ppb AFB1 spike maize, (**b**) 20 ppb AFB1 spiked maize, (**c**) 150 ppb AFB1 spiked maize, and (**d**) 21.8 ppb AFB1 commercially purchased control. Dashed line represents the spiked concentration of AFB1 in maize (n = 9 unless annotated).

While all ELISA kits were able to detect the presence and absence of AFB1, the AgraQuant Kit was found to be the most accurate and precise at 5 ppb, 20 ppb, and 150 ppb with the least variability (see Figure 1). The B-TeZ Kit had a tendency to underestimate AFB1 concentration (mean 2.46 ppb, 10.69 ppb, and 107.60 ppb, respectively), while the Bioscience Kit generally overestimated AFB1 concentration as suggested by the recovery values (mean 5.65 ppb, 31.09 ppb, and 184.72 ppb, respectively). Finally, RidaScreen demonstrated slightly higher accuracy as compared to B-TeZ and Bioscience kits (mean 4.67 ppb, 16.66 ppb, and 136.60 ppb, respectively), but did not outperform AgraQuant. Further analysis of these results is presented in Table S1. Trilogy control sample concentrations were confirmed via HPLC.

**Figure 2:**
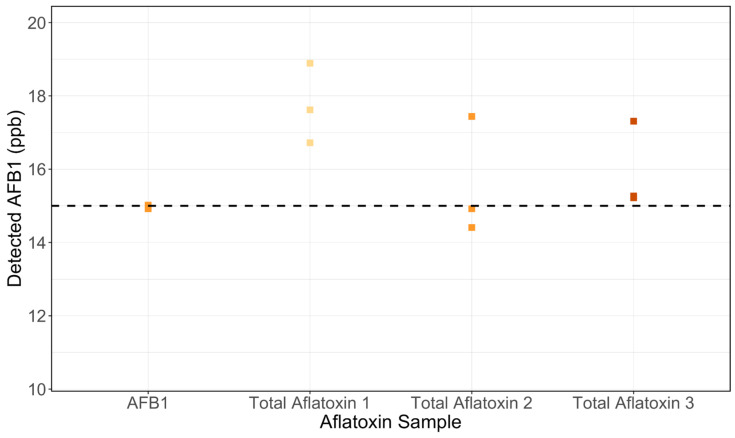
AgraQuant AFB1 Detection with Cross-Reactivity. AgraQuant AFB1 quantification remained robust when challenged with cross-reactivity from AFB_2_, AFG_1_, and AFG_2_. Total aflatoxin 1, 2, and 3 are samples spiked with all four aflatoxins. Dashed line indicates the AFB1 concentration in the AFB1-only spiked sample.

**Figure 3:**
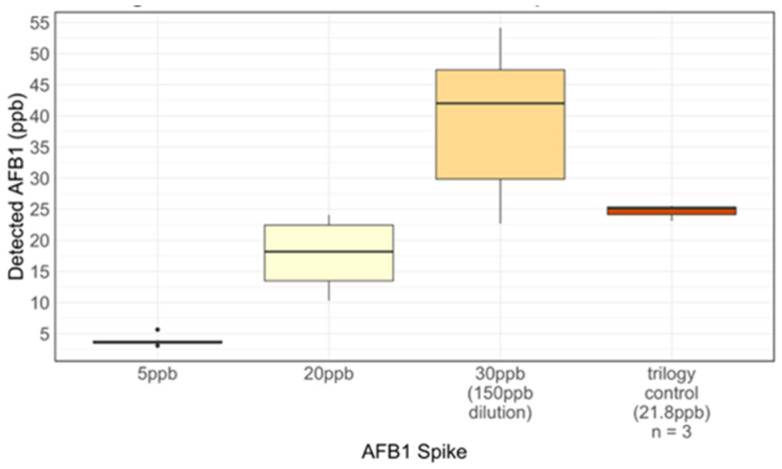
AgraQuant AFB1 Detection in a Tortilla Matrix at Varying Concentrations.

Consequently, the AgraQuant ELISA Kit was chosen to conduct further testing for (1) cross-reactivity in maize to other aflatoxins (AFB2, AFG1, and AFG2) and (2) validity of AFB1 detection in tortillas. The AgraQuant Kit demonstrated minimal cross-reactivity compared to the AFB1-only control with no more than a 17% inflated AFB1 estimate in the presence of the other aflatoxins for 8 of 9 samples (see Figure 2). The AgraQuant Kit retained similar accuracy when testing tortillas, with a slightly higher variation for the 20 ppb and 150 ppb spike-in levels (see Figure 3). Respective means and standard deviations across the 5 ppb, 20 ppb, and 150 ppb tortilla spike-ins and trilogy samples were as follows: 3.78 ± 0.75 ppb, 17.84 ± 5.21 ppb, 196.0 ± 54.06 ppb, and 24.61 ± 1.30 ppb.

### 2.2. Lateral-Flow Assay Test

The lateral flow tests coupled with the image analysis program were performed on a total of 91 lateral flow images from two different lots (or kits). The confusion matrix is depicted in Table 2. Individual lot models performed well with high precision (100% for both) and accuracy (88% and 93% for lot 1 and 2, respectively). However, when combining lots, precision was degraded (76%), while accuracy remained good (84%). Next, the best fitting multiple regression model utilized the mean saturation, median saturation, median saturation of the top 25% saturated pixels, and contrast. For lot 1, the model had an R^2^ value of 0.52, and for lot 2, 0.74 (see Figures S1–S3 for scatter plots of predicted vs. actual values). Unfortunately, combining these two lots resulted in a much poorer R^2^ value of 0.301, potentially due to inter-lot variability.

### 2.3. Barriers to Use

Table 3 highlights the key challenges practitioners in decentralized agricultural systems in low-resource settings face when monitoring AFB1 in supply chains. These include barriers related to costs, power requirements, procurement, and required personnel skills.

For **costs**, simpler methods like the Moisture Reader and BGYF are far more accessible, with capital costs under USD $600 and negligible per-sample costs, making them ideal for low-resource settings. However, these are still out of reach for subsistence farmers. Advanced methods like HPLC and LC-MS, with capital costs up to USD $100,000 and high per-sample expenses, are often impractical without external funding. ELISA and LFA methods occupy a mid-tier but may face challenges in kit costs depending on import requirements. For **power requirements**, battery-operated tools like the Moisture Reader and BGYF are well suited for settings with unreliable electricity. More advanced methods, such as ELISA, HPLC, and LC-MS, require consistent power, adding significant barriers in decentralized regions.

For **procurement**, the Moisture Reader and BGYF benefit from greater local availability, whereas ELISA, LFA, HPLC, and LC-MS rely on more specialized suppliers and potentially imported reagents, creating logistical and financial hurdles. For **skills and uses**, simpler tools like the Moisture Reader and BGYF require minimal training and operate quickly, making them practical for field use. In contrast, ELISA, HPLC, and LC-MS demand extensive training and technical expertise, limiting their feasibility in regions with limited resources. Overall, simple, low-cost methods align well with the realities of low-resource settings, while advanced technologies, despite their accuracy, remain inaccessible due to high costs, power dependency, and technical complexity.

## 3. Discussion

Given the various factors of tropical and subtropical climate, limitations in regulatory frameworks and testing methods, and pre-harvest and post-harvest practices affecting formal and informal supply chains, AFB1 contamination of maize especially challenges low-resource countries. Consequently, increased accessibility to AFB1 testing methods suitable for decentralized, small-holder-driven agricultural economies, prevalent in low-income settings, are necessary. In this study, we identified and addressed the following as pertinent steps towards meeting this need: (1) a comparative AFB1 ELISA kit assessment of accuracy and precision, (2) the application of an LFA image-based algorithm to AFB1 quantification, and (3) the evaluation of barriers-to-use of AFB1 quantification methods in low-resource settings. Four commercial ELISA kits were comparatively evaluated, among which the RomerLabs AgraQuant Aflatoxin B1 2/50 ELISA Kit was found to be a promising screening tool, accurately quantifying maize samples ranging from 5 ppb to 150 ppb AFB1. Further testing on the AgraQuant ELISA Kit minimally altered accuracy when applied to AFB1-contaminated tortillas and natural cross-contamination by the four major aflatoxins altogether. However, with ELISA methods still necessitating expensive and bulky equipment for readings, this study also explored LFAs as a cost-effective option, by creating a 84% accurate imaging algorithm for detection to bypass the expensive purchase of digital dipstick readers. Finally, the barriers-to-use analysis suggested that while low-cost, portable methods like the Moisture Reader and BGYF screening are practical for initial field-level assessments in low-resource settings, their qualitative nature limits broader applicability, necessitating a tiered approach that integrates these tools with more advanced methods, such as ELISA or HPLC, for confirmatory testing and regulatory enforcement.

While previous studies have validated ELISA kits with respect to HPLC for aflatoxin quantification in various types of feedstuffs, evidenced accuracy and repeatability was limited to lower contamination ranges of 2 to 4 ppb [22]. In this study, validating the contamination range of 5 ppb to 150 ppb AFB1 in maize was necessary to comprehensively assess their suitability to test for naturally occurring concentrations of contamination at various stages in the formal and informal supply chain. Therefore, the unexpectedly high variance in performance observed between the AgraQuant, Bioscience, Ridascreen, and B-TeZ ELISA kits may be attributed to differences in the protocols’ detection range compatibility and incubation periods. Accordingly, the low-performance of the B-TeZ ELISA Kit that worsened above 5 ppb may be explained by its narrow detection range of 0.05 ppb to 5 ppb AFB1 [23], while necessary dilutions may introduce additional variability. Shorter assay time and incubation periods, minimizing increased variability due to longer periods of exposure to ambient factors, potentially explain the superior performance of the AgraQuant ELISA Kit [23,24,25,26]. Any inconsistencies in reagent quality across kits likely influence reactivity and color-signal clarity as well. Previous studies validating ELISA kits against HPLC for total aflatoxins and AFB1 also report slightly increased variability, with some findings especially linking ELISA overestimation to matrix effects in contaminated foodstuffs [11,27]. While evidence of variability across ELISA replicates supports its use primarily as a screening tool, other studies substantiate its performance as sufficiently comparable to that of confirmatory tools such as HPLC [27,28]. With robust QA/QC practices, ELISA kits could be a central component in the confirmatory stages of AFB1 monitoring.

Despite the various advantages of ELISA with respect to HPLC in low-income settings, such as affordability as well as ease and speed of use, this immunochemical testing method still poses challenges for field analysis, requiring a costly and large detector and skilled technicians [29]. Consequently, this study aimed to apply the LFA dipsticks in AFB1 detection to bypass the need of both technicians familiar with ELISA workflow and costly detection hardware. For example, while LFAs designed for AFB1 are in the primary stages of validation and have been studied in mediums like milk, these dipsticks nevertheless require the purchase of expensive automated digital scanners [30]. However, existing work attempts to bypass this requirement with accessible detection algorithms using image processing techniques in other fields, including smartphone cameras and processors, which this study emulated for AFB1 detection in maize [31]. While promising, given the robust accuracy in the presence/absence models, further work is needed to improve algorithm capabilities for between lot issues and quantitative estimates.

The barriers-to-use analysis highlights the need for tailored AFB1 monitoring in decentralized, low-resource agricultural systems. These systems, marked by smallholder farming, informal supply chains, and limited regulatory oversight, pose challenges for implementing advanced detection methods like HPLC or LC-MS, which require significant investment, stable power, and skilled technicians [4,13]. Our findings emphasize the utility of simpler, lower-cost methods such as the Moisture Reader and BGYF for preliminary screening along supply chains. However, their reliance on indirect or qualitative indicators limits their role in regulatory enforcement or precise quantification [14]. This underscores the need for multi-tiered strategies combining field-deployable tools, like LFAs, with centralized methods, such as ELISA, for confirmation. Understanding the cost, power, procurement, and skill barriers for each method allows stakeholders to design context-specific programs that bridge formal and informal supply chains and improve public health outcomes [5,10].

Fumagalli and colleagues have presented a useful framework for mycotoxin monitoring focused on centralized supply chains in high-income settings [32]; however, decentralized supply chains in low-resource settings would benefit from further innovation and framework development. In low-resource contexts, a tiered monitoring approach emphasizing moisture readers and BGYF testing for aggressive initial screening is crucial, including, where feasible, at the farm, transport, and market points. Furthermore, instead of costly confirmatory methods like HPLC or LC-MS/MS, validated ELISA or LFA could provide accessible alternative confirmatory methods. This model could be supported by local agricultural guidelines promoted by extension agents. To enhance feasibility, a rotating or “traveling” ELISA lab could provide scheduled, localized confirmatory testing. In shorter supply chains involving farmers, “middlemen” and local buyers, middlemen could integrate moisture monitoring, BGYF screening, or LFAs into their purchasing process, incentivized by a “Verified Seller” program or “Quality Maize Bonus.” Agricultural extension officers at weekly markets could offer voluntary aflatoxin testing with small financial incentives or vouchers to reward good-quality maize. Additionally, market-wide awareness campaigns with flyers, simple guidelines, and success stories can highlight the financial benefits of high-quality, aflatoxin-free maize, fostering community-wide support for these practices.

This study provides valuable insights into AFB1 monitoring methods in decentralized agricultural systems, but several limitations must be noted. First, only one ELISA kit (AgraQuant) was selected for further validation, and testing was restricted to maize and one maize-based food product, tortillas. Expanding validation to a wider range of products is essential to assess its broader applicability. Second, while the LFA relied on a smartphone-based quantification algorithm instead of the standard digital reader, which was unavailable due to cost, the known spiked concentrations and strong algorithm performance provide confidence in the results. Future work could include validation using the digital reader as a benchmark. Third, the HPLC method used a diode array detector (DAD) instead of the more sensitive fluorescence detector (FLD), but this study’s detection limits and robust controls ensured reliable results. Further research should evaluate cost-effective HPLC configurations tailored to low-resource settings.

## 4. Conclusions

This study highlights the critical need for tailored and accessible AFB1 monitoring methods that align with the unique challenges of decentralized agricultural systems, particularly in low-resource settings. By evaluating and comparing ELISA kits, developing an image-based LFA quantification approach, and assessing barriers-to-use across a spectrum of detection methods, we identified practical opportunities for enhancing aflatoxin monitoring. While advanced methods such as HPLC and LC-MS offer unparalleled precision, their prohibitive costs, infrastructure demands, and technical requirements underscore the necessity of integrating simpler, field-adaptable tools into monitoring frameworks. The successful validation of the AgraQuant ELISA kit and the potential of smartphone-based LFA analysis demonstrate the feasibility of cost-effective, scalable solutions that can bridge gaps in accessibility and reliability. Moving forward, efforts should focus on refining these tools to encompass broader matrices and contamination ranges while also addressing systemic barriers through capacity-building initiatives, public-private partnerships, and regulatory alignment. Ultimately, a multifaceted approach combining technological advancements with strategic implementation can strengthen aflatoxin mitigation efforts and safeguard public health in vulnerable communities worldwide.

## 5. Methods

The laboratory work described below, including ELISA, LFA, and HPLC, was conducted at the Arizona State University Biodesign Institute.

### 5.1. ELISA

Four commercial ELISA Kits were compared in their detection of aflatoxin B_1_: (A) AgraQuant Aflatoxin B1 2/50 ELISA Kit (Romer Labs, Getzersdorf, Austria), hereafter referred to as AgraQuant; (B) Aflatoxin B1 (AFB1) ELISA Kit (AFG Bioscience, Northbrook, IL, USA), hereafter referred to as Bioscience; (C) B-TeZ Aflatoxin B1 Kit, (Bio-TeZ, Berlin, Germany), hereafter referred to as BTeZ; and (D) RIDASCREEN Aflatoxin B1 30/15 (R-Biopharm AG, Darmstadt, Germany), hereafter referred to as Ridascreen. A BioTek Synergy HTX Multimode Reader (BioTek Instruments Inc., Winooski, VT, USA) was used for the absorbance plate reading of the ELISA Kits.

Ground dry maize, sieved for homogeneity, was used as a feed sample in the comparative evaluation of aflatoxin B1 (AFB1) detection by commercial ELISA Kits. The dry maize samples used in this study were obtained from Food to Live (Brooklyn, NY, USA). Aflatoxin in Corn Quality Control Material (21.8 ppb—121126, Trilogy Analytical Laboratory, Washington, MI, USA) was used as an internal control. A total of 5 g of ground maize was distributed into 50 mL conical centrifuge tubes for each sample. Excluding negative control samples, the samples were then spiked at known AFB1 concentrations, 5 ppb, 20 ppb, and 150 ppb. Target concentrations were achieved by pipetting 1 µL, 4 µL, and 30 µL of 10 µg/mL aflatoxin B1 in acetonitrile (CTSL-131-5, Trilogy Analytical Laboratory, Washington, MI, USA). The primary comparative analysis of the four kits utilized 9 samples across each concentration with the exception of the 5 ppb AFB1 B-TeZ condition (n = 3).

A 70% methanol solution was prepared and homogenized with a serological pipette in a graduated cylinder. A total of 25 mL of 70% MeOH was distributed into each sample, after which the centrifuge tubes were vigorously shaken for one minute. The maize was allowed to sediment over 10 to 15 min, after which the supernatant was poured into clean conical centrifuge tubes through a funnel lined with Whatman #1 filter paper. The 150 ppb sample was further diluted in a 1:4 ratio to a 30 ppb solution within the ELISA kits’ limits of detection. Each sample extraction yielded between 12 to 15 mL of supernatant. These extracts were tested for viable pH within a range of 6 to 8. The samples and ELISA kits were stored at 4 °C for the duration of the validation study. The ELISA tests were conducted and analyzed as instructed by the respective kit manufacturers, diluting samples with provided buffer solutions and reagents for further sample preparation.

After comparative evaluation of the AFB1 ELISA Kits, the impact of cross-reactivity due to the presence of aflatoxins B_2_, G_1_, and G_2_ on the AgraQuant Aflatoxin B1 2/50 ELISA Kit validity was further investigated. Samples were prepared as described above. Cross-reactivity was tested in triplicate across three different spike-ins of 15 ppb AFB1, with the exception of the AFB1 control (n = 2). Cross-reactive samples were further spiked with respective 3 ppb AFB_2_, 3 ppb AFG_2_, and 10 ppb AFG_1_ before the addition of 70% MeOH. The AgraQuant Aflatoxin B1 2/50 ELISA Kit was also tested for viability across maize mediums, using dry maize tortillas instead of dry maize (hard maize), with subsequent steps proceeding identically. Tortilla analysis was conducted by using nine samples for each AFB1 spike-in concentration.

In order to calibrate the absorbance plate readings of the ELISA tests, manufacturer instructions and provided sets of known standards were used to create log-linear standard curves, scaling absorbance against AFB1 concentration (see Table S2). Running standard curves along with each set of samples for within-day repeatability, Bioscience and B-TeZ standard curves were generated in triplicate, while four AgraQuant standard curves and one Ridascreen standard curve were generated (see Figures S4 and S5).

The raw data were first imported into Microsoft Excel, organized and cleaned to generate standard curves, estimate AFB1 concentrations, and calculate mean and standard deviation across spike-in levels, ELISA kits, and maize medium. The standard curves for each commercial kit were modified as necessary to meet selection criteria of an absolute R-value of >0.99 by trimming outliers while ensuring that each curve consisted of at least 4 data points. These descriptive statistics were imported into R to generate scatterplots and boxplots for comparative visualization.

### 5.2. Lateral Flow Assay “Dipstick” Test

A total of 50 g of ground maize spiked with AFB1 at a concentration of 21.8 ppb (Trilogy Analytical Laboratory, Washington, MO, USA) was used for extraction. The Reveal Q+ MAX for Aflatoxin (Neogen, Lansing, MI, USA) MAX 1-G50 aqueous extraction packet was added to the container, along with 250 mL of deionized water. This solution was shaken for 3 min and subsequently filtered into a clean vial using Grade 1 filter paper (Zenpore, Fotan Hong Kong, New Territories). In order to create samples of different concentrations to test with the dipsticks, the 21.8 ppb solution was diluted with deionized water to a range of specific AFB1 concentrations: 0 ppb (deionized water only), 0.5 ppb, 1 ppb, 2 ppb, 2.5 ppb, 3.5 ppb, 4 ppb, 5 ppb, 7 ppb, and 10 ppb. The limit of detection for the LFA kit was 3 ppb, therefore we increased the number of tests conducted between 2 and 4 ppb to initially develop a presence/absence model. We then included additional concentrations between 0 and 10 ppb to test a linear predictive model as well as further refine the presence/absence model. The dipstick tests were conducted according to the protocol of the manufacturer.

Following the test procedure, each test strip was photographed on both black and white backgrounds using the 48-megapixel rear camera on an iPhone 14 (Apple Inc., Cupertino, CA, USA). Images were captured at the highest resolution available and saved in RAW format using the default camera application under consistent lighting conditions. The total number of test trips used were N = 50. Imaging repetition (</=2 replicas per image) produced a subsequent sample pool consisting of twenty 0 ppb, twelve 0.5 ppb, twelve 1 ppb, four 2 ppb, four 2.5 ppb, five 3.5 ppb, four 4 ppb, six 5 ppb, twelve 7 ppb, and twelve 10 ppb images.

In order to quantify AFB1 based on dipstick test images, a computer program was developed to compare the color saturation levels of the control lines with the toxicity indicator lines on the tests (see Figure S6). The primary step of this program involved image preprocessing. First, the images were cropped to consist entirely of the dipstick test, positioning the control line and toxicity indicator line at the top and bottom of the image, respectively. Normalization of the cropped images was then performed through background subtraction, pixel-level subtraction, and scaling (Python 3.13.0, OpenCV library). In background subtraction, bounding boxes were manually drawn around the blank portion of the dipstick test, and the average pixel values for this region were calculated. Subsequent pixel-level subtraction involved deduction of the mean pixel values of the blank region from those in the entire image, accentuating the visibility of both the control and toxicity indicator lines to ease later analysis. Lastly, the images were scaled to a standardized intensity range of 0–255 using min-max normalization.

Following image preprocessing, bounding boxes were manually drawn around the control and toxicity indicator lines. The program calculated average saturation values for each of these regions of interest and generated a ratio of toxicity indicator saturation to control saturation. This ratio-based approach was necessary to minimize the effect of discrepancies between dipstick tests and lots, improving the reliability of the toxin concentration estimations.

The process was performed on a total of 91 dipstick images from 2 different lots. A confusion matrix (e.g., true positive, false negative) was generated and a multiple linear regression model was estimated utilizing the ratio of control line values to toxin indicator line values as its parameters. Statistical analysis was conducted using Microsoft Excel v2411.

### 5.3. HPLC

To test recovery with HPLC, the ELISA extraction procedure was carefully followed with ground maize spiked to 100 ppb and an internal control of Aflatoxin in Corn Quality Control Material (233.0 ppb—121123, Trilogy Analytical Laboratory, Washington, Missouri). Extracts were centrifuged at 10,000 rpm for 10 min. Supernatant was syringe filtered into an amber glass HPLC vial using a sterile 3 mL syringe with a 0.2 µm PTFE filter attached. Samples were then run on an Agilent 1260 Infinity II HPLC (Agilent Technologies, Santa Clara, CA, USA). The HPLC method was previously described by Glesener et al., 2024, but is briefly described here for clarity [33]. The method ran for 8 min using an isocratic mobile phase (50% water, 40% methanol, and 10% acetonitrile) set at 0.800 mL/min on an Agilent Eclipse XDB-C18 4.6 × 150, 3.5 µm Rapid Resolution column at 40 °C with a diode array detector.

### 5.4. Evaluation of Barriers-to-Use

To evaluate key barriers-to-use for these methods, we adapted agricultural technical evaluation approaches described by Dibbern et al. and Baumuller et al. [34,35]. Specifically, we evaluated these monitoring methods based on the following criteria: cost (capital and operational), power requirements, procurement needs, and skills/uses including required training, amount of personnel needed, and use of equipment for other applications. We provide a synthesized breakdown of each of these areas for each testing method within the context of decentralized agricultural systems.

## Figures and Tables

**Table 1:** Common AFB1 testing methods.

	Method	Application, Purpose, USDA Approval	Applicable Portions of Supply Chain	Pros	Cons
Moisture Reader	Indirect, presumptive method associating moisture content that promotes fungal growth with AFB1 presence.	Screening Risk assessment Indirect approval	Post-harvest: storage, processing, transport, and sale. [14] Informs <14% moisture content storage conditions. [18]	Rapid and non-destructive sampling, bypasses user skills. No prior sample preparation.	Purported indirect method. Lacks accurate quantification. Device cost USD $500. (+/−)
BGYF	Presumptive method of hyperspectral imaging based on UV light absorption and subsequent emission of blue-green-yellow visible fluorescence by AFB1 metabolites [19].	Screening Fungal contamination check Approved for screening	Market and household applications for end users with small-scale sorting and removal of contaminated maize kernels [17]. Post-harvest monitoring to inform storage and mechanical drying methods [20].	Rapid and non-destructive sampling for real-time application [19]. Portable and low-cost screening optimizing profit and food resources for local and subsistence farmers and rural households, respectively [17].	Relatively high Type I and II errors detecting intermediate metabolites i.e., kojic acid [19]. Lower predictive precision and accuracy supporting qualitative use [19].
LFA	Immunochemical dipstick method for qualitative presence/absence, quantified with reader or algorithm.	Screening * Rapid aflatoxin detection Approved for screening	Market and household applications for end users with rapid on-site screening [12].	Rapid, portable, and user friendly. Qualitative and quantitative use.	High cost of the portable digital reader needed to quantify dipsticks. Lower sensitivity with high lower-limits of detection.
ELISA	Colorimetric enzyme immunoassay quantifying AFB1 using microplate absorbance reader.	Confirmatory Quantitative aflatoxin test Approved for compliance	Post-harvest monitoring and quality control.	High throughput using 96-well microplate. Relatively rapid preparation and analysis time.	Variability between commercial kits. Applicability to food products (beyond grain). Requires trained laboratory personnel. High cost of the lab-based ELISA plate reader.
HPLC	Column chromatographic technique allowing for compound separation and aflatoxin quantification within sample.	Confirmatory Precise aflatoxin quantification Fully approved	Post-harvest: quality assurance for food products and in laboratory settings.	Highly accurate and precise quantitative analysis.	Requires skilled technicians and expensive equipment. Time consuming and limited on-site analysis options. Standard AFB1 detector (FLD) is not widely used beyond AFB1 molecule.
LC-MS	Combination of tandem mass spectroscopy and liquid chromatography, separating, identifying, and quantifying multiple compounds including AFB1 in a sample.	Confirmatory High-sensitivity quantification Fully approved	Post-harvest: quality assurance for food products and in laboratory settings.	Highly accurate with low LOD due to high sensitivity and specificity.	Requires skilled technicians and expensive equipment. Time consuming and inapplicable to on-site analysis.

* If standards are consistent and testing ranges are adequate, it can be used for confirmatory reporting.

**Table 2:** Confusion matrix of lateral flow test analysis.

	TPR	FPR	TNR	FNR	Precision	Accuracy	F1
Lot 1 Model (n = 74)	0.78	0.00	1.00	0.22	1.00	0.88	0.88
Lot 2 Model (n = 17)	0.86	0.00	1.00	0.14	1.00	0.93	0.92
Combined Model (N = 91)	0.90	0.21	0.79	0.10	0.76	0.84	0.82

TPR, true positive rate; FPR, false positive rate; TNR, true negative rate; FNR, false negative rate.

**Table 3:** Barriers-to-use for commonly used AFB1 testing methods.

	Costs **	Power	Procurement	Skills and Uses *
Moisture Reader	Capital: USD $400–600 (reader). Per sample: USD $0.	Battery (for reader).	Commonly available.	Training: simple. Personnel: one person for 30 s. Other uses: moisture content of other grains.
BGYF	Capital: USD $100–300 (black light, box, glasses). Per sample: USD $0.	Battery (for black light).	Commonly available.	Training: simple. Personnel: one person for 2 min. Other uses: none.
LFA	Capital (with reader): USD $5000. Capital (without reader): USD $0. Per sample:	Reader requires electrical outlet (read time only X seconds).	Kits offered by just a few suppliers. Imports may be tricky. Basic lab supplies commonly available.	Training: moderate. Personnel: one person for 10 min. Other uses: none.
ELISA	Capital: USD $3,000–10,000 (plate reader). Per sample: USD $10–15.	Reader (and computer, if applicable) requires electrical outlet (read time 20 s).	Kits offered by just a few suppliers. Imports may be tricky or expensive. Basic lab supplies commonly available. Some restrictions on specific reagents (e.g., methanol).	Training: extensive. Personnel: one person for 2 h (dependent on # of samples). Other uses: yes, plate reader is compatible with many ELISA kits.
HPLC	Capital: USD $30,000 (used)–80,000 (new). Per sample: USD $25–35.	Machine, detector, and computer require electrical outlets (run time can be 24+ h).	Reagents available, but imports may be tricky or expensive. Larger parts (columns, detectors, etc.) may be difficult to obtain.	Training: extensive. Personnel: one person for 4 h (dependent on # of samples). Other uses: yes, can quantify many other compounds with relevant reagents.
LC-MS	Capital: USD $80,000 (used)–250,000 (new). Per sample: USD $50–100.	Machine, detector, and computer require electrical outlets (run time can be 24+ h).	Reagents available, but imports may be tricky or expensive. Larger parts (columns, detectors, etc.) may be difficult to obtain.	Training: extensive. Personnel: one person for 4 h (dependent on # of samples). Other uses: yes, can quantify many other compounds with relevant reagents.

* Includes training, number of personnel required, and other valuable applications of the device; ** per sample costs do not include labor.

## Data Availability

The majority of the original contributions presented in this study are included in the article/Supplementary Material. Additional data and code can be provided with guidance via inquiries directed to the corresponding author.

## Supplementary Materials

The following supporting information can be downloaded at https://www.mdpi.com/article/10.3390/toxins17010037/s1: Table S1: Summarized Comparative Results of AFB1 Detection Across Four ELISA Kits; Figure S1: Scatter plot and linear model of actual vs predicted AFB1 values for Lot 1; Figure S2: Scatter plot and linear model of actual vs predicted AFB1 values for Lot 2; Figure S3: Scatter plot and linear model of actual vs predicted AFB1 values for combined lots; Table S2: AFB1 standards provided by the ELISA kit manufacturers; Figure S4: Results of the mean standard curves for the ELISA kits; Figure S5: Results of the individual standard curves for the ELISA kits; Figure S6: Example image of the LFA dipsticks used for image analysis. Supplemental Data File S1: LFA Training Data.
