# Supplementary material for: Evaluating Methods for Aflatoxin B1 Monitoring in Selected Food Crops Within Decentralized Agricultural Systems
Source: Toxins (Basel). 2025 Jan 14;17(1):37. doi: 10.3390/toxins17010037 (PMC11769523; doi:10.3390/toxins17010037)
Supplement: Supplementary file 1 [file toxins-17-00037-s001.zip › AFB1-monitoring_supplementary_material_final.pdf]

Supplemental Material

Evaluating Methods for Aflatoxin B1 Monitoring in Food Crops within Decentralized Agricultural Systems

Table S1. Summarized Comparative Results of AFB1 Detection Across Four ELISA Kits

ELISA Kit	Concentration (ppb)	Mean ± SD	Recovery (%)
**AgraQuant**	5	5.34 ± 0.53	106.87
	20	19.16 ± 1.29	95.81
	150	150.75 ± 13.72	100.50
	21.8 (trilogy control)	20.82 ± 0.16	95.50
**Bioscience**	5	5.65 ± 1.21	113.02
	20	31.09 ± 5.61	155.44
	150	184.72 ± 52.21	123.14
	21.8 (trilogy control)	38.61 ± 4.43	177.11
**B-Tez**	5	2.46 ± 2.20	49.13
	20	10.69 ± 4.90	53.45
	150	107.60 ± 79.77	71.73
	21.8 (trilogy control)	28.06 ± 17.93	128.72
**Ridascreen**	5	4.67 ± 0.94	93.33
	20	16.66 ± 6.03	83.28
	150	136.60 ± 54.57	91.06
	21.8 (trilogy control)	20.63 ± 11.26	94.66

Figure S1. Scatter plot and linear model of actual vs predicted AFB1 values for Lot 1

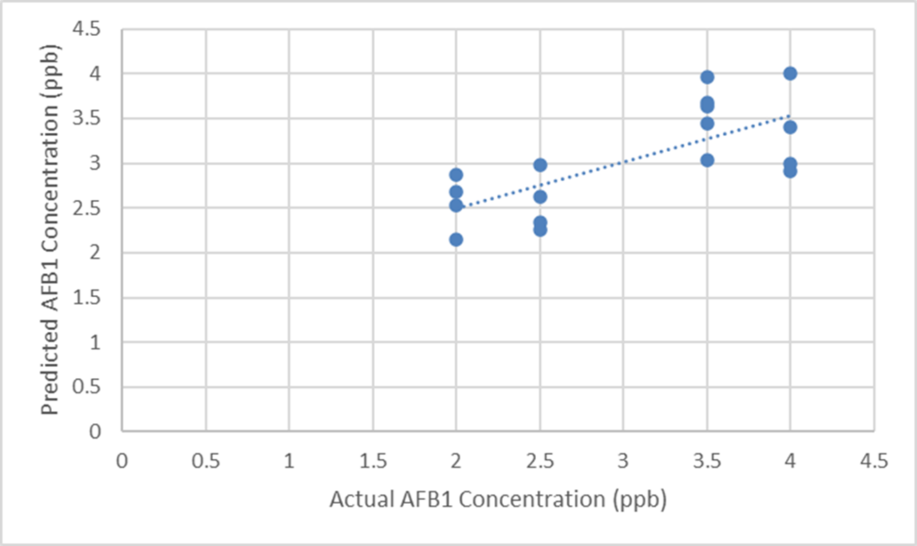

Figure S2. Scatter plot and linear model of actual vs predicted AFB1 values for Lot 2

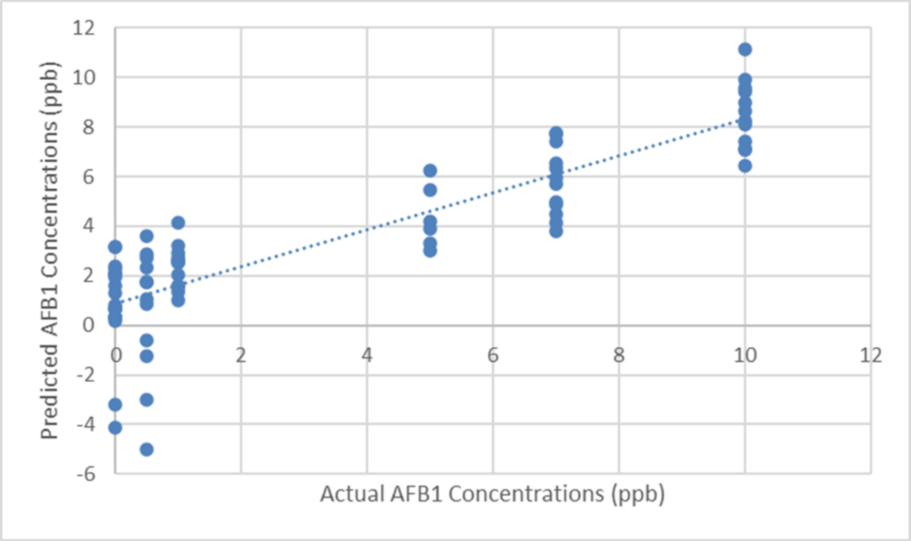

Figure S3. Scatter plot and linear model of actual vs predicted AFB1 values for combined lots

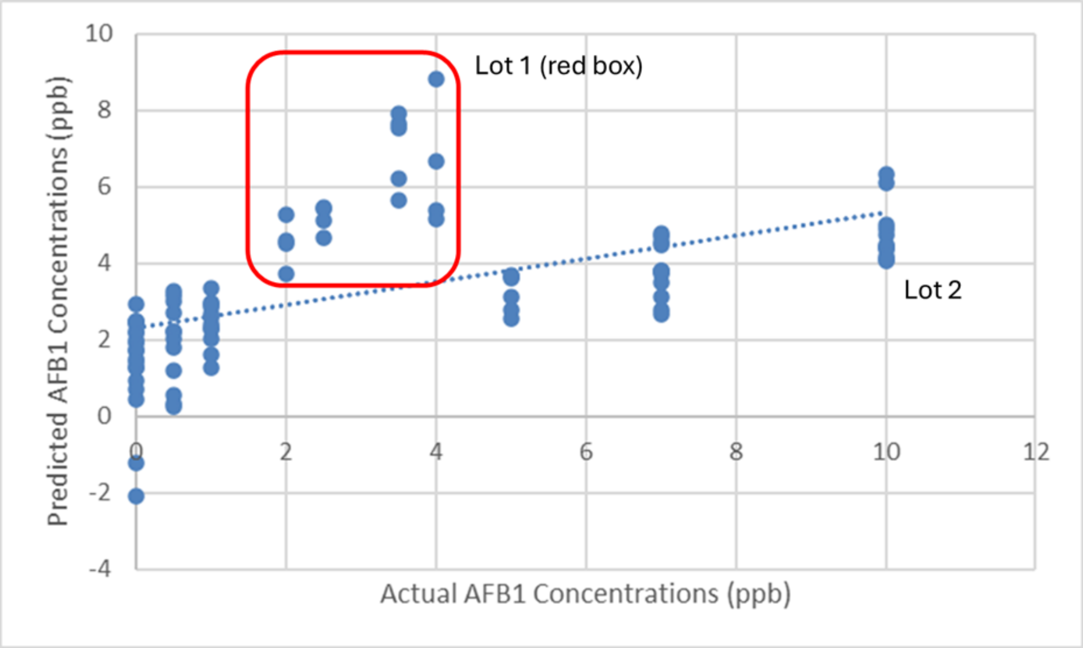

Table S2. AFB1 standards provided by the ELISA kit manufacturers

Standard Solutions	Agraquant	Ridascreen	B-TeZ	Bioscience
Std 1	0 ppb	0 ppb	0 ppb	0 ppb
Std 2	2ppb	1 ppb	0.05 ppb	0.01 ppb
Std 3	5 ppb	5 ppb	0.10 ppb	0.03 ppb
Std 4	20 ppb	10 ppb	0.25 ppb	0.09 ppb
Std 5	50 ppb	20 ppb	0.5 ppb	0.27 ppb
Std 6		50 ppb	1.2 ppb	0.81 ppb
Std 7				100 ppb

Figure S4. Results of the mean standard curves for the ELISA kits

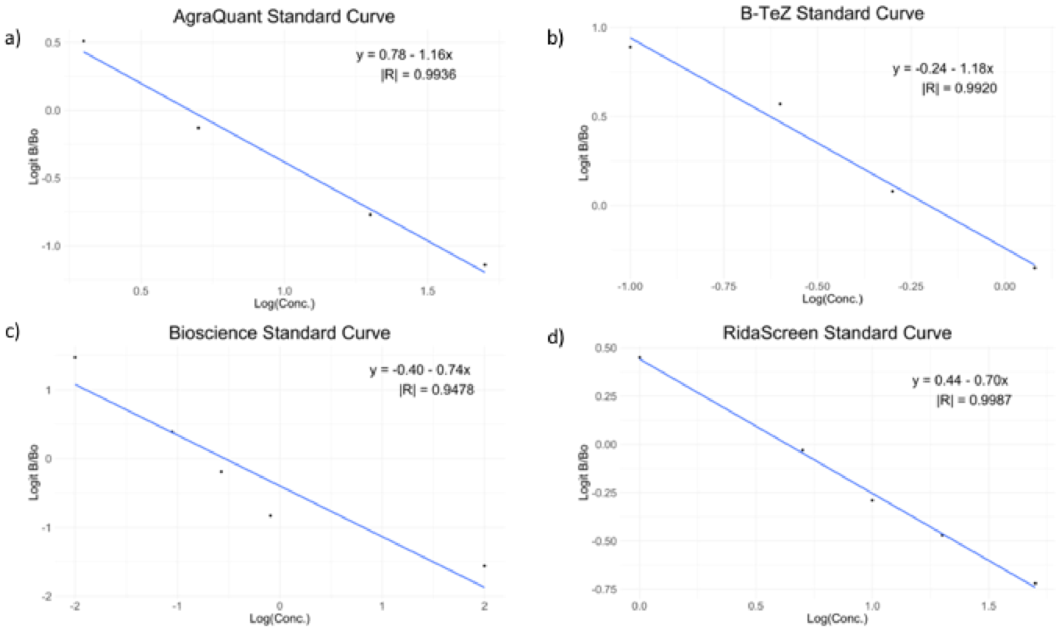

Figure S5. Results of the individual standard curves for the ELISA kits

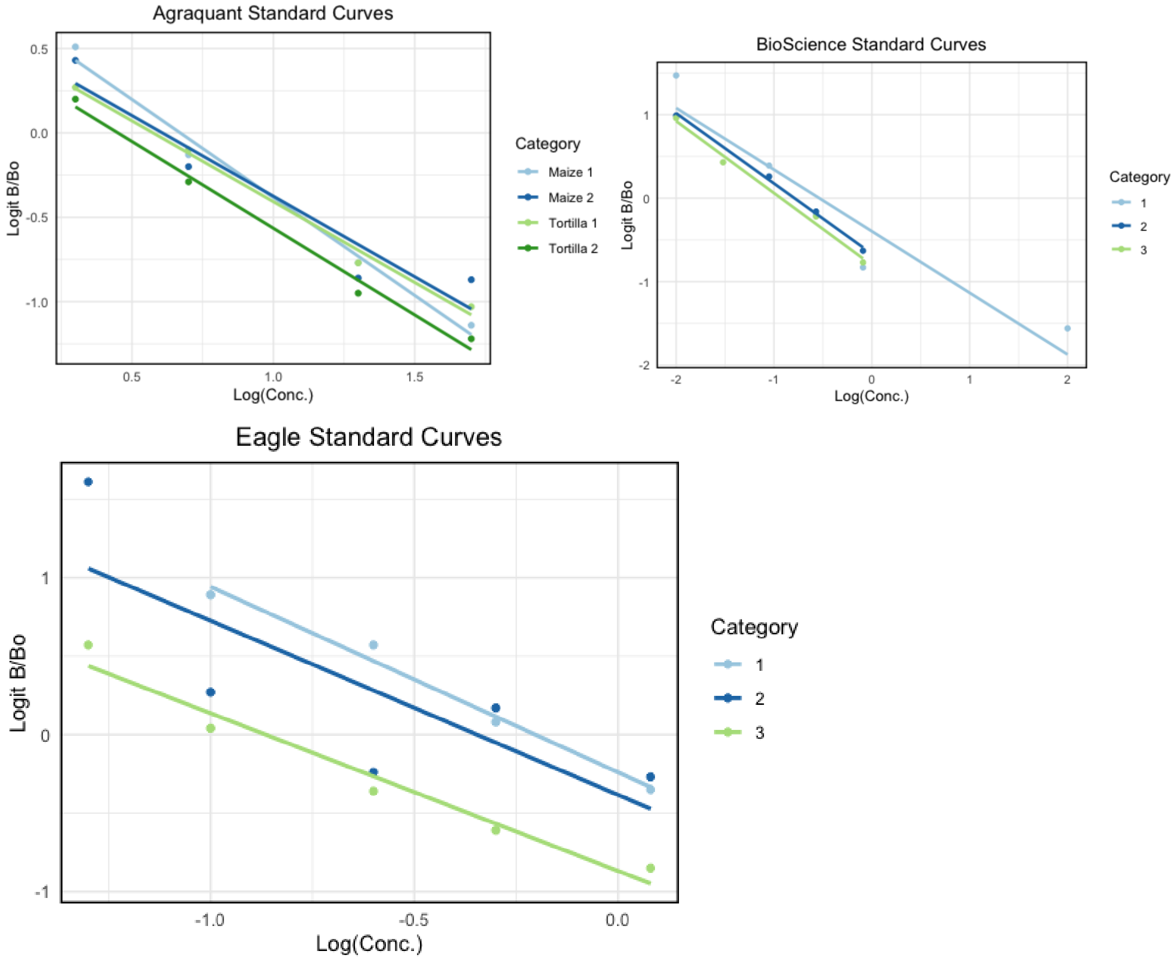

Figure S6. Example image of the LFA dipsticks used for image analysis

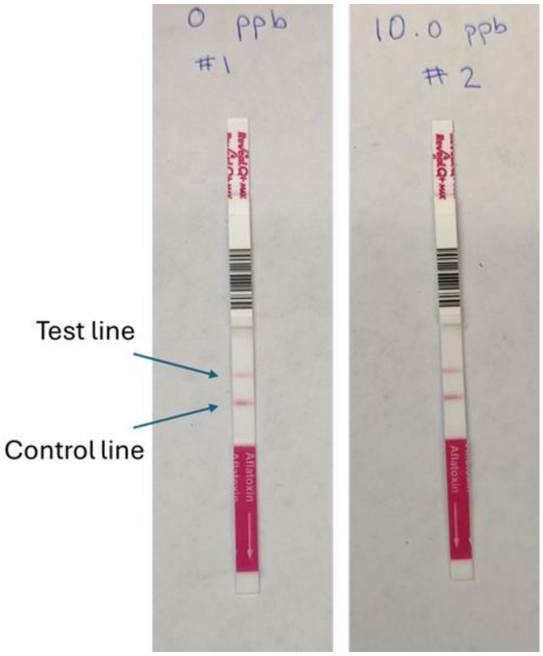

Even at 0 ppb the test line appears, requiring an algorithm to decipher positive vs negative.
